# The Role of Vitamin D and Omega-3 PUFAs in Islet Transplantation

**DOI:** 10.3390/nu11122937

**Published:** 2019-12-03

**Authors:** Marco Infante, Camillo Ricordi, Nathalia Padilla, Ana Alvarez, Elina Linetsky, Giacomo Lanzoni, Alessandro Mattina, Federico Bertuzzi, Andrea Fabbri, David Baidal, Rodolfo Alejandro

**Affiliations:** 1 Diabetes Research Institute (DRI) and Clinical Cell Transplant Program, Miller School of Medicine, University of Miami, Miami, FL 33136, USA; cricordi@med.miami.edu (C.R.); nxp467@miami.edu (N.P.); axa383@med.miami.edu (A.A.); glanzoni@med.miami.edu (G.L.); dbaidal@med.miami.edu (D.B.); ralejand@med.miami.edu (R.A.); 2 Department of Systems Medicine, University of Rome “Tor Vergata”, 00133 Rome, Italy; andrea.fabbri@uniroma2.it; 3 Diabetes Research Institute (DRI) and Cell Transplant Center, cGMP Cell Processing Facility, Miller School of Medicine, University of Miami, Miami, FL 33136, USA; elinetsk@med.miami.edu; 4 Diabetes and Islet Transplantation Unit, Department of Diagnostic and Therapeutic Services, IRCCS-ISMETT (Istituto Mediterraneo per i Trapianti e Terapie ad alta specializzazione), UPMC, 90127 Palermo, Italy; amattina@upmc.it; 5 Diabetology Unit, Niguarda Hospital, 20162 Milan, Italy; federico.bertuzzi@ospedaleniguarda.it

**Keywords:** type 1 diabetes, T1D, islet transplantation, NOD mice, graft survival, immune tolerance, autoimmunity recurrence, allograft rejection, vitamin D, omega-3 PUFAs

## Abstract

Recurrence of autoimmunity and allograft rejection represent major challenges that impact the success of islet transplantation. Despite the remarkable improvements achieved in immunosuppression strategies after the publication of the Edmonton protocol, long-term data of intra-hepatic islet transplantation show a gradual decline in beta-cell function. Therefore, there is a growing interest in the investigation of novel, safe and effective anti-inflammatory and immunomodulatory strategies able to promote long-term islet graft survival and notable improvements in clinical outcomes of islet transplant recipients. Vitamin D has been shown to exert anti-inflammatory and immunomodulatory effects. Pre-clinical studies investigating the use of vitamin D and its analogs (alone or in combination with immunosuppressive agents and/or other anti-inflammatory agents, such as omega-3 polyunsaturated fatty acids) showed beneficial results in terms of islet graft survival and prevention of recurrence of autoimmunity/allograft rejection in animal models of syngeneic and allogeneic islet transplantation. Moreover, epidemiologic studies demonstrated that vitamin D deficiency is highly prevalent after solid organ transplantation (e.g., heart, liver or kidney transplantation). However, studies that critically assess the prevalence of vitamin D deficiency among islet transplant recipients have yet to be conducted. In addition, prospective studies aimed to address the safety and efficacy of vitamin D supplementation as an adjuvant immunomodulatory strategy in islet transplant recipients are lacking and are therefore awaited in the future.

## 1. Introduction 

Type 1 diabetes (T1D) is a chronic, organ-specific autoimmune disease characterized by the progressive destruction of pancreatic beta cells, which ultimately results in lifelong dependence on exogenous insulin. Pancreatic islet transplantation is a beta-cell replacement therapy that has showed efficacy in improving glycemic control, reducing glycemic variability, abolishing hypoglycemia and improving quality of life in patients with T1D complicated by severe hypoglycemia and hypoglycemia unawareness [1,2,3,4,5]. Despite the remarkable improvements achieved in anti-inflammatory and immunosuppression strategies after the publication of the Edmonton protocol, which is based on a glucocorticoid-free immunosuppressive regimen [6], long-term data of intra-hepatic islet transplantation show a progressive decline in beta-cell function over time [7]. Also, Ryan et al. showed that only a minority of T1D patients (approximately 10%) who underwent islet transplantation were able to maintain insulin independence over a 5-year follow-up period, despite ongoing evidence of persistent graft function, as indicated by restored C-peptide secretion [8].

The progressive graft dysfunction observed over the years after transplantation is due to several factors, such as recurrence of autoimmunity, allograft rejection and metabolic exhaustion [9,10]. Additionally, the site of transplantation might represent a problem. Currently, allogenic islet cells are infused intra-portally. One of the limitations of an intravascular site (e.g., intraportal) for islet transplantation is represented by the instant blood-mediated inflammatory reaction (IBMIR), which is triggered when islets are exposed to blood in the immediate post-transplant period [11,12]. IBMIR leads to platelet consumption, activation of complement and coagulation systems and islet infiltration by leukocytes, resulting in significant impairment to islet morphology and function [11,13]. Islet graft injury mediated by pro-inflammatory cytokines released from islet-infiltrating immune cells represents another important mechanism of early islet graft dysfunction. Moreover, intra-hepatic islet transplantation leads to thrombosis and liver ischemia due to islet entrapment in the liver sinusoids and subsequent activation of Kupffer cells and sinusoidal endothelial cells [14,15]. In animal models, these events have been shown to result in a loss of approximately 60% of transplanted islets, which has been attributed to apoptosis and necrosis occurring during the first days post-transplantation [13,16]. Also, the intra-hepatic site is associated with the direct exposure of islet graft to orally ingested immunosuppressive drugs, which can exert toxic effects on islet function before undergoing the first-pass metabolism [10,17].

Prevention of IBMIR, recurrence of autoimmunity and allograft rejection represent major challenges in clinical islet transplantation. Therefore, there is growing interest in the investigation of novel, safe and effective strategies aimed to counteract inflammatory responses in the immediate post-transplant period, restore immune tolerance, prevent allograft rejection and promote long-term islet graft survival in order to improve clinical outcomes of islet transplant recipients.

Herein, we will summarize the results from pre-clinical studies that evaluated the use of vitamin D and its analogs (alone or in combination with immunosuppressive agents and/or other anti-inflammatory agents, such as omega-3 polyunsaturated fatty acids) as an adjuvant immunomodulatory strategy in animal models of syngeneic and allogeneic islet transplantation. Therefore, this paper will aim to define a rationale for investigation of vitamin D and/or omega-3 polyunsaturated fatty acids (PUFAs) in clinical pancreatic islet transplantation for treatment of T1D. Furthermore, we also provide data on vitamin D and omega-3 PUFA supplementation in islet transplant recipients with long-term allograft function who completed last follow-up visit at our Institution.

## 2. Vitamin D

In addition to the well-established regulation of calcium/phosphate metabolism and bone homeostasis, vitamin D exerts several anti-inflammatory and immunomodulatory effects [18,19]. Vitamin D is a steroid hormone that is mainly produced from human skin upon sunlight exposure [20], although it can be also obtained from a few dietary sources, such as vegetables (e.g., yeast and fungi) or animal-derived foods (e.g. cod liver oil and fatty fish) that contain vitamin D2 (ergocalciferol) and vitamin D3 (cholecalciferol), respectively [21,22]. Of note, vitamin D-fortified milk represents the main source of vitamin D both for children and adults in the US [23,24]. Under sunlight exposure, 7-dehydrocholesterol present in keratinocytes is converted into vitamin D3. Vitamin D3 undergoes a first hydroxylation catalyzed by the liver enzyme vitamin D-25-hydroxylase, resulting in the synthesis of 25-hydroxyvitamin D3 (the major circulating metabolite of vitamin D), which is finally converted into the biologically active form 1,25-dihydroxyvitamin D3—1,25(OH)2D3, referred to as calcitriol—in the kidneys by 1α-hydroxylase enzyme [25]. Calcitriol exerts its effects on classical vitamin D target tissues (intestine, bone, kidney and parathyroid glands) through the activation of vitamin D receptor (VDR), a nuclear receptor that belongs to the steroid receptor transcription factor family [25].

Notably, functional VDR has also been detected in almost all immune cells, particularly antigen presenting cells (APCs) and T cells [26,27]. Additionally, 1α-hydroxylase is expressed by murine and human APCs [28,29] and it can be induced upon interferon-gamma (IFN-γ) and lipopolysaccharide (LPS) stimulation, although this phenomenon is highly species-specific and occurs in human and pig monocytes/macrophages but not in murine APCs [30,31]. Calcitriol exerts several effects on both innate and adaptive immune system, resulting in induction of immune tolerance and activation of anti-inflammatory pathways. Notably, calcitriol: (i) promotes macrophage differentiation and activation and enhances macrophage antimicrobial activity by stimulating the local production of defensins [32], (ii) inhibits the synthesis of pro-inflammatory cytokines by monocytes and macrophages [33], (iii) reduces macrophage surface expression of major histocompatibility complex (MHC)-class II molecules, thus decreasing the macrophage antigen-presentation and T-cell stimulatory ability [28,34], (iv) promotes the shift of macrophage polarization from M1 phenotype (pro-inflammatory or “classically activated” macrophages) towards M2 phenotype (anti-inflammatory or “alternatively activated” macrophages) [35], (v) modulates the differentiation and function of dendritic cells (DCs), rendering them more tolerogenic and reducing their antigen-presenting capacity [36,37,38,39,40], (vi) up-regulates regulatory T cells (Tregs) [41], (vii) promotes the shift of T cells from an “effector” towards a “regulatory” and anti-inflammatory phenotype by increasing T-helper (Th) 2 cells and reducing Th1 and Th17 cell differentiation [42,43,44].

Several pre-clinical studies conducted in non-obese diabetic (NOD) mice showed that calcitriol and its analogs are able to prevent or arrest the progression of autoimmune diabetes and insulitis [45,46,47,48,49]. On the other hand, clinical studies investigating the use of vitamin D as an immunomodulatory strategy aimed to preserve residual beta-cell function in T1D presented inconclusive results, probably due to heterogeneity in duration of follow-up and vitamin D doses and formulations [50]. However, pre-clinical evidence suggests a potential role of vitamin D and its analogs in prolonging islet graft survival by preventing autoimmunity recurrence and allograft rejection (discussed later in the text).

## 3. Omega-3 PUFAs

Omega-3 PUFAs are the precursors of a series of anti-inflammatory lipid mediators called “specialized pro-resolving lipid mediators” (SPMs), which include different families of chemically and physiologically distinct mediators (lipoxins, resolvins, protectins and maresins) [51]. These mediators are able to counteract neutrophil infiltration, reduce pro-inflammatory cytokine expression and promote efferocytosis of apoptotic neutrophils and bacterial clearance [52,53]. In particular, eicosapentaenoic acid (EPA) is the precursor of E-series resolvins (RvE1, RvE2, RvE3), whereas docosahexaenoic acid (DHA) is the precursor of three distinct families of SPMs that include D-series resolvins (RvD1, RvD2, RvD3, RvD4), protectins (PD1, also referred to as neuroprotectin—NPD1—when formed in the central nervous system) and maresins (MaR1) [51].

Endres et al. [54] first reported that dietary supplementation with 5 g/day of EPA and DHA in healthy human volunteers was able to significantly reduce the in vitro LPS-induced production of pro-inflammatory cytokines (interleukin-1 and tumor necrosis factor) by peripheral blood mononuclear cells. Moreover, potential synergistic anti-inflammatory properties exerted by the co-administration of vitamin D and omega-3 PUFAs have been shown in different clinical settings [55,56]. Importantly, Kagohashi et al. [57] showed that a diet with a low omega-6/omega-3 essential fatty acids (EFA) ratio was associated with significantly longer survival in NOD mice when administered within 6 days after the onset of overt diabetes. More recently, a study conducted by Bi et al. [58] in NOD mice demonstrated that intervention with dietary omega-3 PUFAs (EPA and DHA) decreased the incidence of severe insulitis and diabetes, reduced the proportion of Th1 and Th17 cells, increased the proportion of Th2 cells and Tregs, and reduced the expression of several pro-inflammatory cytokines, such as IFN-γ, interleukin (IL)-17, IL-6 and TNF-α. Consistent with the findings observed in NOD mice, the authors also found that EPA and DHA were able to increase Th2 cell populations and reduce Th1 cell populations in human peripheral blood mononuclear cells isolated from T1D patients. Conversely, NOD mice fed with a diet enriched with the omega-6 PUFA arachidonic acid (AA) showed a significant increase in the proportion of Th1 cells, an exacerbated ratio of Th1/Th2 cells, along with an increase in the proportion of Th17 cells [58]. These findings may be partly explained by the fact that AA is the precursor of pro-inflammatory eicosanoids [51,59]. Accordingly, AA/EPA ratio—which is a surrogate marker of the omega-6/omega-3 ratio—has been suggested as an inflammatory biomarker [60,61,62], with lower levels potentially being associated with a reduced magnitude of inflammation [60] and beneficial effects in different clinical settings [63,64,65,66,67,68], including T1D [69,70,71]. In particular, three case reports showed that co-administration of cholecalciferol and high dose omega-3 PUFAs (55–70 mg of EPA and DHA/kg body weight/day) was able to preserve residual beta-cell function and promote partial clinical remission in children with newly diagnosed T1D [69,70,71]. Therefore, we are conducting a phase I/II clinical trial (POSEIDON, Pilot Study of Omega-3 and Vitamin D in High-Dose in Type I Diabetic Patients; ClinicalTrials.gov Identifier: NCT03406897) aimed to evaluate whether 12-month co-supplementation with cholecalciferol plus omega-3 PUFAs (150 mg of EPA and DHA/kg body weight/day) is able to halt autoimmunity and preserve residual beta-cell function in both pediatric and adult subjects with newly diagnosed and established T1D [72].

## 4. Syngeneic Islet Transplantation

Mathieu et al. first showed that calcitriol and cyclosporine A (CyA, an immunosuppressive drug that selectively inhibits calcineurin) exert synergistic effects on the inhibition of human T-cell proliferation in vitro, thus suggesting calcitriol as a potential dose-reducing agent for CyA in immunosuppressive regimens [73]. Indeed, the authors also investigated the efficacy of the 20-epi calcitriol analog (KH1060, referred to as lexacalcitol) in preventing the recurrence of autoimmunity following syngeneic islet transplantation in NOD mice [73,74]. Interestingly, compared to high doses of KH1060 or CyA administered as monotherapies, low doses of KH1060 and CyA were well-tolerated and more effective in maintaining graft function and prolonged graft survival after discontinuation of treatment [73,74]. Moreover, analysis of cytokine expression at the graft level revealed that NOD mice treated with KH1060 plus CyA displayed significantly lower levels of IL-12 and IFN-γ, along with significantly higher levels of IL-4 compared to those treated with KH1060 or CyA alone [75]. These data indicate that a combination of KH1060 and CyA might prolong syngeneic islet graft survival by promoting a shift of T cell phenotype from Th1 to Th2 in the immediate post-transplant period. Another study conducted in NOD mice transplanted with syngeneic islets demonstrated that treatment with high doses of CyA or KH1060 was able to significantly prolong islet survival, when compared to vehicle (peanut oil)-treated controls, although recurrence of autoimmunity occurred shortly after the treatment was discontinued [76]. NOD mice treated with low doses of both drugs also exhibited a significantly longer graft survival compared to controls. More importantly, 5 out of 13 of these mice were still normoglycemic 60 days post-transplant and 4 of them did not show disease recurrence for more than 15 days after the treatment was discontinued. Histological analysis revealed a diminished degree of immune cell infiltration and beta-cell destruction in grafts of NOD mice treated with combination therapy compared to controls [76].

Van Etten et al. [77,78] evaluated the ability of the vitamin D3 analog TX527 to prevent recurrence of autoimmunity and promote graft survival in NOD mice following syngeneic islet transplantation. The authors demonstrated that the combination of TX527 and IFN-β or CyA was associated with a significantly prolonged islet graft survival, compared to vehicle (peanut oil), monotherapy or IFN-β plus CyA combination therapy. No major adverse effects were observed. In addition, cytokine mRNA analysis of NOD islet grafts performed 6 days after transplantation revealed a significant reduction in IL-2, IL-12 and IFN-γ in mice treated with TX527 plus IFN-β or CyA, compared to vehicle-treated mice. This evidence suggests that the combination therapy approach may inhibit the Th1 pathway [78]. In keeping with these findings, Baeke et al. [79] demonstrated that low doses of TX527, CyA and anti-CD3 monoclonal antibody exerted synergistic effects in delaying recurrence of autoimmune diabetes after syngeneic islet transplantation in NOD mice. Of note, triple-combination therapy that consisted of TX527, CyA and anti-CD3 monoclonal antibody was well-tolerated and resulted in significant prolongation of islet graft survival compared to anti-CD3 monotherapy or dual therapy with anti-CD3 plus CyA. Moreover, triple-combination therapy was demonstrated to be superior to anti-CD3 monotherapy in decreasing islet graft infiltration by CD4+ and CD8+ T cells, reducing pro-inflammatory cytokine expression and increasing the frequency of Tregs in blood, spleen and kidney draining lymph nodes [79]. However, it is worth noting that vitamin D analogs cannot be considered nutrients but they are drugs who have been developed for hyperproliferative disorders in order to reduce the occurrence of adverse calcemic side effects (hypercalcemia and hypercalciuria) potentially caused by supraphysiological doses of calcitriol [80]. In this regard, both KH1060 and TX527 have different pharmacokinetic and pharmacodynamic properties compared to calcitriol, including a reduced ability to cause hypercalcemia and hypercalciuria [80].

**Figure 1:**
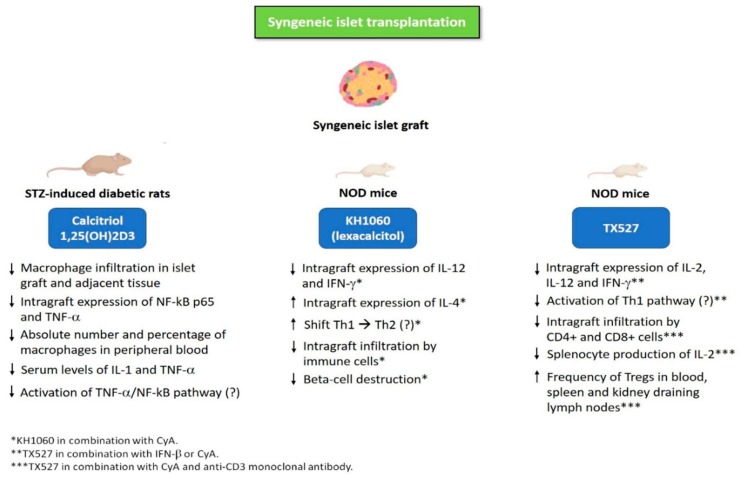
Mechanisms underlying the protective effects of vitamin D and its analogs (alone or in combination with immunosuppressive agents) against recurrence of autoimmunity and graft failure in animal models of syngeneic islet transplantation. Abbreviations: CyA, cyclosporine A; IFN-β, interferon-beta; IFN-γ, interferon-gamma; IL-1, interleukin-1; IL-2, interleukin-2; IL-4, interleukin-4; IL-12, interleukin-12; NF-kB p65, nuclear factor kB p65; NOD mice, non-obese diabetic mice; STZ, streptozotocin; Th1, T-helper 1 cell; Th2, T-helper 2 cell; TNF-α, tumor necrosis factor-alpha; Tregs, regulatory T cells.

Jiao et al. [81] showed that calcitriol significantly prolonged islet graft survival in streptozotocin (STZ)-induced diabetic rats following syngeneic intraportal islet transplantation. Notably, calcitriol led to a reduced macrophage infiltration in both islet graft and adjacent tissue, which was also accompanied by a decreased intra-graft expression of nuclear factor kB (NF-kB) p65 and TNF-α, along with lower serum levels of IL-1 and TNF-α compared to control group. These results seem to suggest that calcitriol may prolong syngeneic islet graft survival by reducing inflammation via decreased activation of TNF-α/NF-kB pathway and reduced macrophage recruitment in transplanted graft [81]. Figure 1 illustrates the mechanisms underlying the protective effects of vitamin D and its analogs (alone or in combination with immunosuppressive agents) against recurrence of autoimmunity and graft failure in animal models of syngeneic islet transplantation. Table 1 summarizes the studies evaluating the use of vitamin D and its analogs (alone or in combination with immunosuppressive agents) in animal models of syngeneic islet transplantation.

## 5. Allogeneic Islet Transplantation

Some studies investigated the effects of vitamin D and its analogs in animal models of allogeneic islet transplantation to assess whether they may prevent allograft rejection and/or prolong allograft survival. Gregori et al. [82] showed that a 30-day calcitriol and mycophenolate mofetil (MMF) treatment of STZ-induced diabetic mice, transplanted with allogeneic islets, significantly prolonged islet graft survival compared to mice treated with calcitriol or MMF alone. Mice with functioning grafts for over 70 days after transplantation were injected intraperitoneally with donor spleen cells to look at transplantation tolerance. Interestingly, MMF and calcitriol combination therapy resulted in significantly higher resistance to islet graft rejection, compared to MMF or calcitriol monotherapy [82]. In another study [83], the same authors demonstrated that a short-term treatment with MMF and calcitriol resulted in induction of tolerance to islet allografts in STZ-induced diabetic recipient mice. Of note, MMF and calcitriol combination therapy inhibited the peri-graft recruitment of macrophages and DCs, which both displayed a tolerogenic phenotype consisting of down-regulated expression of CD40, CD80 and CD86 costimulatory molecules, along with a remarkably reduced IL-12 secretion. Importantly, down-regulation of costimulatory molecules persisted for over 100 days after treatment discontinuation. Additionally, MMF and calcitriol combination therapy also increased the frequency of CD4+CD25+ regulatory T cells in the spleen and in the kidney lymph nodes draining the islet graft. This cell population was shown to transfer long-term transplant tolerance in naïve syngeneic recipient mice, accompanied by a decreased frequency of IFN-γ-producing CD4+ and CD8+ cells. These findings suggest that CD4+CD25+ regulatory T cells may play an important role in tolerance induction and prevention of islet allograft rejection in vivo [83]. Ferreira et al. [39] demonstrated that murine bone marrow-derived DCs exposed to calcitriol display a more tolerogenic phenotype compared to control cells, resulting in a reduced expression of MHC II co-stimulatory molecules CD80 and CD86. The authors also showed that transfer of calcitriol-modulated DCs was able to prevent hyperacute graft rejection in alloxan-induced diabetic mice transplanted with allogeneic islets, although long-term graft survival did not differ compared to control animals (recipients who did not receive any immunomodulatory treatment).

**Figure 2:**
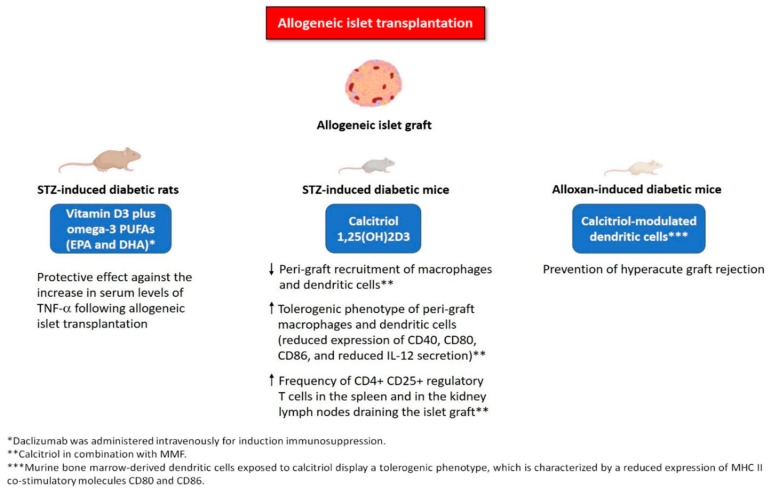
Mechanisms underlying the protective effects of vitamin D and its analogs (alone or in combination with immunosuppressive agents and/or other anti-inflammatory agents, such as omega3 PUFAs) against allograft rejection and graft failure in animal models of allogeneic islet transplantation. Abbreviations: EPA, eicosapentaenoic acid; DHA, docosahexaenoic acid; IL-12, interleukin-12; MHC, major histocompatibility complex; MMF, mycophenolate mofetil; PUFAs, polyunsaturated fatty acids; STZ, streptozotocin; TNF-α, tumor necrosis factor-alpha.

With regard to omega-3 PUFAs, Gurol et al. [84] showed that combination therapy of vitamin D3 plus EPA and DHA was able to counteract the increase in serum levels of TNF-α following allogeneic islet transplantation in STZ-induced diabetic rats. This effect might have been mediated by the synergistic anti-inflammatory actions of vitamin D and omega-3 PUFAs. In keeping with this hypothesis, bone marrow-derived mouse DCs exposed to resolvin E1 (RvE1) have been shown to induce apoptosis of activated CD4+ T cells [85]. Lund et al. [86] demonstrated that RvE1 significantly reduced the LPS-induced upregulation of pro-inflammatory cytokines (IL-8, monocyte chemotactic protein-1 and tissue factor), as well as the cytokine-induced apoptosis in human pancreatic islets in vitro. Intriguingly, BLT1 (a cell surface receptor for RvE1) was found to be expressed in human islets. RvE1 was also able to significantly lower the ADP/ATP ratio, although it had no effect on insulin secretion [86]. Figure 2 illustrates the mechanisms underlying the protective effects of vitamin D and its analogs (alone or in combination with immunosuppressive agents and/or other anti-inflammatory agents) against allograft rejection and graft failure in animal models of allogeneic islet transplantation. Table 2 summarizes the studies evaluating the use of vitamin D and its analogs (alone or in combination with immunosuppressive agents and/or other anti-inflammatory agents) in animal models of allogeneic islet transplantation. 

## 6. Role of Vitamin D in Clinical Solid Organ Transplantation

To date, there is dearth of epidemiologic studies that address vitamin D deficiency among islet transplant recipients. However, several studies addressing this subject have been conducted among solid organ transplant recipients [87]. Notably, vitamin D deficiency is highly prevalent and severe during the immediate post-transplant period following solid organ transplantation (e.g., heart, liver or kidney transplantation) and persists in long-term allograft recipients [87,88,89,90,91,92,93,94]. This may be due to several factors, namely: (i) inadequate vitamin D dietary intake or supplementation after transplantation [91,95]; (ii) reduced sun exposure (usually recommended to organ transplant recipients in order to prevent the risk of skin cancer related to long-term immunosuppression) [96,97]; and (iii) increased vitamin D catabolism or reduced vitamin D hydroxylation induced by glucocorticoids and/or immunosuppressive drugs [87,98,99,100,101]. Therefore, vitamin D deficiency should be promptly diagnosed and treated in organ transplant recipients, since it can potentially result in secondary hyperparathyroidism and bone loss [102,103], which can be further exacerbated by the detrimental skeletal effects mediated by immunosuppressive drugs [104]. Courbebaisse et al. [100] showed that high-dose vitamin D3 (100,000 IU every 2 weeks) during the first months after kidney transplantation is safe and effective in increasing serum 25(OH)D levels (above 30 ng/mL) and reducing parathyroid hormone levels. Moreover, the Kidney Disease Improving Global Outcomes (KDIGO) guidelines and the National Kidney Foundation/Kidney Disease Outcomes Quality Initiative (NKF-KDOQI) suggest that vitamin D deficiency in kidney transplant recipients should be corrected using the same therapeutic strategies recommended for the general population in order to reduce the risk of detrimental skeletal consequences (hyperparathyroidism, bone loss, fractures) [105,106].

As previously discussed, vitamin D exerts antimicrobial, anti-inflammatory and immunomodulatory effects [32,107,108,109]. Therefore, the importance of vitamin D treatment after transplantation (including islet transplantation) may go beyond its beneficial skeletal effects. The rationale for vitamin D use in transplant recipients may rely on its potential protective action against opportunistic infections, recurrence of autoimmunity and allograft rejection [110]. Notably, animal studies suggest that calcitriol and its analogs are able to prevent acute allograft rejection and prolong allograft function following kidney [111], liver [112,113] and heart [114] transplantation. Moreover, calcitriol treatment has been retrospectively associated with lower episodes of acute allograft rejection and reduced glucocorticoid requirements after kidney transplantation in human subjects [115,116]. Conversely, the use of prednisone and some immunosuppressive agents (e.g., mycophenolate sodium, tacrolimus) has been significantly associated with higher prevalence of vitamin D deficiency among kidney transplant recipients [117]. Additionally, a prospective study conducted in kidney transplant recipients showed that patients treated with calcitriol exhibited a decreased expression of HLA-DR and co-stimulatory molecules (CD28, CD86 and CD40) in peripheral blood leukocytes [118], thus providing a potential mechanistic explanation for the role of calcitriol in prolongation of allograft survival and prevention of allograft rejection in solid organ transplant recipients. Despite this evidence, there is paucity of prospective studies that address the role and mechanism of action of vitamin D in human transplant recipients in general and islet cell transplant recipients in particular.

## 7. Discussion

Pre-clinical evidence suggests that vitamin D and its analogs (alone or in combination with immunosuppressive medications or other anti-inflammatory agents, such as omega-3 PUFAs) are safe and effective immunomodulatory agents in animal models of syngeneic and allogeneic islet transplantation. In animal models of syngeneic islet transplantation, vitamin D and its analogs appear to play a role in the prevention of autoimmunity and disease recurrence. In addition, vitamin D and its analogs have also been shown to promote islet engraftment, prevent allograft rejection and prolong islet graft survival in animal models of allogeneic islet transplantation. These effects may be explained, at least in part, by the afore mentioned anti-inflammatory and immunomodulatory properties of vitamin D. In particular, vitamin D may have a role in the prevention of IBMIR, which is associated with a significant islet graft loss in the immediate post-transplant period. Moreover, it is worth noting that TNF-α is one of the major pro-inflammatory cytokines released upon the activation of Kupffer cells following intrahepatic islet transplantation [14], thus justifying the introduction of pharmacological blockade of TNF-α signaling pathway in human islet transplant recipients [119,120]. Interestingly, calcitriol has been shown to significantly suppress TNF-α expression by human monocytes [121]. In addition, calcitriol may also prevent beta-cell apoptosis, which appears to represent an important contributor to the loss of transplanted islets during the immediate post-transplant period in murine models of syngeneic islet transplantation [122]. Riachy et al. demonstrated that calcitriol was able to induce and maintain high levels of the anti-apoptotic protein A20 [123] and to counteract the expression of the pro-apoptotic transmembrane cell surface receptor Fas in human pancreatic islets exposed to pro-inflammatory cytokines (IL-1β, IFN-γ and TNF-α) in vitro [124]. These results suggest that calcitriol may exert a protective effect on the apoptotic signaling pathways triggered by pro-inflammatory cytokines in beta cells.

Importantly, several epidemiologic studies showed that severe vitamin D deficiency is highly prevalent in solid organ transplant recipients. However, epidemiologic studies on prevalence of vitamin D deficiency among islet transplant recipients have not yet been conducted and are therefore awaited. Pre-clinical data presented earlier provide a strong rationale for clinical studies to tackle the role of vitamin D in islet transplant recipients. Key questions to be addressed should focus on the ability of vitamin D to:

- prevent IBMIR and promote islet engraftment in the immediate post-transplant period
- prevent acute allograft rejection
- prevent or delay recurrence of autoimmunity by restoring immune tolerance
- promote long-term islet graft survival and function
- modulate the reduction in the administered dose of immunosuppressive drugs and associated adverse events (e.g., opportunistic infections, bone loss and beta-cell toxicity)

We evaluated serum 25(OH)D levels and plasma AA/EPA ratio in 17 islet transplant recipients with long-term allograft function currently being followed at our Institution (Diabetes Research Institute, Clinical Cell Transplant Program, University of Miami Miller School of Medicine; Table 3). At the last follow-up visit (2019; mean duration of islet allograft function after islet transplantation: 11.1 ± 8.4 years), mean serum 25(OH)D levels were 51.0 ± 7.0 ng/mL (reflecting an optimal vitamin D status [125]), whereas mean plasma AA/EPA ratio values were 31.3 ± 12.3. Among these 17 islet transplant recipients, 15 subjects were on cholecalciferol supplementation (mean total daily dose—1626 IU), whereas 5 subjects were on omega-3 PUFA supplementation (mean total daily dose—2000 mg; supplementation with an EPA/DHA ratio of 2:1). Among patients on omega-3 PUFA supplementation, AA/EPA ratio values were 3.7 ± 1.7 (Table 3). At the present time, we cannot determine if the supplementation with vitamin D and/or omega-3 PUFAs may have contributed to the maintenance of long-term islet allograft function.

With regard to omega-3 PUFA, only a few studies have evaluated their anti-inflammatory properties in human pancreatic islets in vitro and in animal models of allogeneic islet transplantation [84,86]. However, the potential ability of omega-3 PUFAs to improve long-term islet allograft survival and function will be partly addressed by the ongoing phase I/II clinical trial “Allogeneic Islet Cells Transplanted Onto the Omentum” (ClinicalTrials.gov Identifier: NCT02213003), which is testing the omentum as a novel extra-hepatic site for islet transplantation. In this trial, high dose omega-3 PUFAs (up to 6750 mg EPA and 3375 mg DHA/day) will be administered in the peri-transplant period and continued thereafter as an adjuvant anti-inflammatory therapy to improve long-term islet allograft survival, in addition to pegylated granulocyte-colony stimulating factor (G-CSF), glucagon-like peptide (GLP)-1 receptor agonist exenatide and supplemental oxygen therapy via nasal cannula.

## 8. Conclusions

In conclusion, epidemiologic studies evaluating the prevalence of vitamin D deficiency, and large prospective studies investigating the safety and efficacy of vitamin D therapy (alone or in addition to other anti-inflammatory agents, such as omega-3 PUFAs) as a novel immunomodulatory strategy in islet transplant recipients, are warranted at this time. Moreover, prospective studies in islet transplant recipients will be necessary in order to evaluate the influence of vitamin D status and omega-6/omega-3 ratio on long-term islet allograft function.

## Figures and Tables

**Table 1:** Summary of the studies evaluating the use of vitamin D and its analogs (alone or in combination with immunosuppressive agents) in animal models of syngeneic islet transplantation. Abbreviations: CyA, cyclosporine A; IFN-β, interferon-beta; IFN-γ, interferon-gamma; IL, interleukin; MMF, mycophenolate mofetil; MST, mean survival time; NF-kB, nuclear factor kB; NOD mice, non-obese diabetic mice; STZ, streptozotocin; TNF-α, tumor necrosis factor-alpha; Tregs, regulatory T cells.

Study Treatment	Study Treatment Duration	Animal Model	Main Findings	References
KH1060 (Lexacalcitol) *	Treatment was initiated the day before transplantation and continued until 60 days after transplantation	NOD mice receiving syngeneic islets under the kidney capsule	Low doses of KH1060 (0.5 μg/kg/twice daily) and CyA (7.5 mg/kg/day) were well tolerated and more effective compared to high doses of KH1060 (1 μg/kg/twice daily) or CyA (15 mg/kg/day) administered as monotherapies. MST of graft after islet transplantation: KH1060 + CyA group, 62 days; high dose KH1060, 55 days; high dose CyA, 58 days. Cytokine profile expression in islet grafts revealed significantly lower levels of IL-12 and IFN-γ, along with significantly higher levels of IL-4 in NOD mice treated with KH1060 plus CyA compared to those treated with KH1060 or CyA alone.	[73,74,75]
KH1060 (Lexacalcitol) *	Treatment was initiated the day before transplantation and continued until disease recurrence or 60 days after transplantation	NOD mice receiving syngeneic islets under the kidney capsule	MST of graft after islet transplantation: high dose CyA (15 mg/kg/day), 60 ± 26 days; high dose KH1060 (1 μg/kg/2 days), 50 ±15 days; low doses of CyA (7.5 mg/kg/day) plus KH1060 (0.5 μg/kg/2 days), 48 ± 28 days. MST of graft was significantly longer in all the three groups compared to vehicle (peanut oil)-treated controls.	[76]
TX527 **	Treatment was initiated the day before transplantation and continued until day 20 (CyA and IFN-β) or day 30 (TX527) after transplantation	NOD mice receiving syngeneic islets under the kidney capsule	MST of graft after islet transplantation: TX527 (5 μg/kg/day) plus IFN-β (1 × 10^5^ IU/day), 62 ± 20 days; TX527 (5 μg/kg/day) plus CyA (7.5 mg/kg/day), 31 ± 12 days. MST of graft was significantly longer in mice on TX527 plus IFN-β or CyA compared to mice treated with vehicle, monotherapy or IFN-β plus CyA. Mice treated with TX527 plus IFN-β or CyA exhibited significantly reduced graft levels of IL-2, IL-12 and IFN-γ compared to vehicle (peanut oil)-treated controls, as assessed by cytokine mRNA analysis of islet grafts performed 6 days after transplantation.	[77,78]
TX527 **	TX527 and CyA were administered from day 1 until day 60 after transplantation, whereas anti-CD3 monoclonal antibody was administered from day 0 until day 4 after transplantation	NOD mice receiving syngeneic islets under the kidney capsule	Mice receiving triple-combination therapy with TX527 (10 μg/kg every 2 days, day 1 until day 60) plus CyA (5 mg/kg per day, day 1 until day 60) and anti-CD3 monoclonal antibody (2.5 μg/day, days 0–4) showed a significantly longer islet graft survival (MST, 79.5 ± 18.6 days) compared to those receiving anti-CD3 monotherapy (MST, 24.8 ± 7.3 days) and dual therapy with anti-CD3 plus CyA (MST, 25.5 ± 12.4 days). Histology of the transplanted islets revealed that grafts of mice treated with triple-combination therapy were more preserved and less infiltrated by CD4+ cells and effector/memory phenotype CD8+ T cells (on day 21 after transplantation). Mice receiving triple-combination therapy showed significantly increased frequency of Tregs in blood, spleen and kidney draining lymph nodes, compared to untreated (control mice) and anti-CD3-treated mice. Importantly, Tregs isolated from mice receiving triple-combination therapy maintained intact suppressive capacity in vivo, as supported by the fact that they significantly delayed diabetes in the NOD Scid transfer model. Anti-CD3 monotherapy led to increased production of TNF-α, IL-5, IL-21 and IL-10, but the upregulation of these cytokines was abrogated by the triple-combination therapy with anti-CD3 plus TX527 and CyA. Moreover, triple-combination therapy significantly reduced IL-2 production by splenocytes compared to anti-CD3 monotherapy.	[79]
Calcitriol	Calcitriol was administered from day 1 until day 20 after transplantation	Sprague-Dawley STZ-induced diabetic rats receiving syngeneic intraportal islet transplantation	Rats receiving calcitriol (5 mg/day by intraperitoneal injection) exhibited a significantly improved islet graft survival compared to control group (propylene glycol administered by intraperitoneal injection): 50% of recipients in the control group maintained a functioning graft for 14 days, whereas 80% of calcitriol-treated recipients remained euglycemic for at least 14 days. Histology revealed that calcitriol-treated mice exhibited a reduced macrophage infiltration in both islet graft and adjacent tissue 7 days after transplantation. At day 7 after transplantation, the absolute number and percentage of macrophages in peripheral blood were significantly lower in calcitriol group compared to control group. Moreover, calcitriol down-regulated the increase in serum levels of IL-1 and TNF-α compared to the control group. Western blot showed that graft expression of NF-kB p65 and TNF-α was significantly lower in calcitriol-treated mice compared to the control group.	[81]

* KH1060 (Lexacalcitol) is a 20-epi analog of 1,25(OH)2D3 (calcitriol); ** TX527 is a vitamin D3 analog.

**Table 2:** Summary of the studies evaluating the use of vitamin D and its analogs (alone or in combination with immunosuppressive agents and/or other anti-inflammatory agents, such as omega-3 PUFAs) in animal models of allogeneic islet transplantation. Abbreviations: DCs, dendritic cells; DHA, docosahexaenoic acid; EPA, eicosapentaenoic acid; IL, interleukin; MMF, mycophenolate mofetil; MST, mean survival time; PUFAs, polyunsaturated fatty acids; STZ, streptozotocin; TNF-α, tumor necrosis factor-alpha.

Study Treatment	Study Treatment Duration	Animal Model	Main Findings	References
Calcitriol in combination with MMF	MMF and/or calcitriol were administered from the day before transplantation and continued until day 30 after transplantation	BALB/c STZ-induced diabetic mice receiving allogeneic islets under the kidney capsule (pancreatic islets were isolated from C57BL/6 (B6) mice)	MMF (100 mg/kg/day) and calcitriol (5 μg/kg/three times a week) combination therapy was associated with significantly longer islet graft survival compared to MMF or calcitriol alone (% of graft survival 70 days after transplantation: 85%, 52%, 48%, respectively). MMF and calcitriol combination therapy was associated with significantly higher resistance to islet graft rejection in comparison to MMF or calcitriol alone (% of graft survival 100 days after transplantation: 72.2%, 33.3%, 52.7%, respectively).	[82]
Calcitriol in combination with MMF	MMF and/or calcitriol were administered from the day before transplantation and continued until day 30 after transplantation	BALB/c STZ-induced diabetic mice receiving allogeneic islets under the kidney capsule (pancreatic islets were isolated from C57BL/6 (B6) mice)	MMF (100 mg/kg/day) and calcitriol (5 μg/kg/three times a week) combination therapy inhibited the peri-graft recruitment of macrophages and DCs and decreased IL-12 secretion. MMF plus calcitriol increased the frequency of CD4+CD25+ regulatory T cells in the spleen and in the kidney lymph nodes draining the islet graft. These cells were able to transfer long-term transplant tolerance in naïve syngeneic recipient mice (up to 40 days).	[83]
Calcitriol-modulated DCs	Transplant recipient mice received three intravenous transfers of calcitriol-modulated murine DCs on days −10, −3 and 0 before transplantation	C57BL/6 alloxan-induced diabetic mice receiving allogeneic islets under the kidney capsule (pancreatic islets were isolated from BALB/c donor mice). Bone marrow cells were harvested from C57BL/6 mice and subsequently induced to differentiate into mature DCs (10 day-culture). The in vitro DC generation was performed in the absence (control DCs) or presence (10^−8^ M) of calcitriol (calcitriol-modulated DCs). In order to perform the DC transfer experiment in the islet allotransplantation model, DCs were pulsed during the last 48 hours of culture with BALB/c islet antigen (BALB/c islet antigen-loaded control DCs).	5 out of 7 recipient mice receiving calcitriol-modulated DCs before islet allotransplantation did not experience hyperacute graft rejection, that was instead observed in all 4 mice receiving BALB/c islet antigen-loaded control DCs. Islet allograft survival was not consistently prolonged in mice receiving calcitriol-modulated DCs compared to mice who did not receive any immunomodulatory treatment (untreated group): MST, 11.4 ± 2.2 days vs. 9.0 ± 1.0 days, respectively.	[39]
Vitamin D3 plus omega-3 PUFAs (EPA and DHA)	Vitamin D3 and/or omega-3 PUFAs were administered on days 0, 1 and 2 after transplantation. Daclizumab was administered intravenously for induction immunosuppression, at a dose of 0.05 mg/kg body weight before transplantation (day 0) and on days 1 and 2 after transplantation.	STZ-induced diabetic Wistar albino rats receiving allogeneic intraportal islet transplantation	Vitamin D3 (5 μg/kg) plus EPA and DHA (7 mg/kg) significantly reduced the increase in serum levels of TNF-α at days 1 and 2 after transplantation compared to control group and rats treated with vitamin D3 or omega-3 PUFAs alone.	[84]

**Table 3:** Demographic characteristics and data on vitamin D supplementation, omega-3 PUFA supplementation, serum vitamin D levels and plasma AA/EPA ratio of islet transplant recipients with long-term allograft function who completed last follow-up visit (2019) at Diabetes Research Institute (Clinical Cell Transplant Program, University of Miami Miller School of Medicine). Abbreviations: 25(OH)D, 25-hydroxyvitamin D; AA, arachidonic acid; BMI, body mass index; DHA, docosahexaenoic acid; EPA, eicosapentaenoic acid; IU, international units; PUFA, polyunsaturated fatty acid; SD, standard deviation.

*n*	17
Gender	12 females, 5 males
Mean age ± SD (years)	55.2 ± 4.9
Mean BMI ± SD (kg/m^2^)	22.1 ± 0.4
Mean duration of graft function ± SD (years)	11.1 ± 8.4
Mean serum 25(OH)D levels ± SD (ng/mL)	51.0 ±7.0
Mean plasma AA/EPA ratio ± SD	31.3 ± 12.3
Vitamin D users *	*n* = 15
Mean total daily dose of vitamin D (IU/day)	1626 IU
Mean daily dose of vitamin D (IU/kg/day)	28.4
Omega-3 PUFA users (EPA and DHA) **	*n* = 5 ***
Mean total daily dose of omega-3 PUFAs (mg/day)	2000
Mean daily dose of omega-3 PUFAs (mg/kg/day)	33.5
Mean plasma AA/EPA ratio values ± SD among omega-3 PUFA users	3.7 ± 1.7

* All subjects were on cholecalciferol (vitamin D3); ** Omega-3 PUFA supplementation consisted of an EPA/DHA ratio of 2:1; *** 4 out of 5 omega-3 PUFA users were also on vitamin D supplementation (vitamin D and omega-3 PUFA co-supplementation).
